# Editorial: JAK inhibition in autoimmune and inflammatory diseases

**DOI:** 10.3389/fimmu.2022.1120281

**Published:** 2022-12-28

**Authors:** Jean-Baptiste Telliez, Massimo Gadina, Kamran Ghoreschi, Olli Silvennoinen, Francesca Romana Spinelli

**Affiliations:** ^1^ Inflammation and Immunology, Pfizer, Cambridge, MA, United States; ^2^ Translational Immunology Section, National Institute of Arthritis and Musculoskeletal and Skin Diseases, National Institutes of Health, Bethesda, MD, United States; ^3^ Department of Dermatology, Venereology and Allergology, Charité—Universitätsmedizin Berlin, Berlin, Germany; ^4^ Faculty of Medicine and Health Technology, Tampere University, Tampere, Finland; ^5^ Dipartimento di Scienze Cliniche, Internistiche, Anestesiologiche e Cardiovascolari—Reumatologia, Sapienza Universitá di Roma, Roma, Italy

**Keywords:** autoimmune diseases, JAK - STAT signaling pathway, JAK, STAT (signal transducer and activator of transcription), inflammatory disease

The journey to characterize the central role of JAK/STAT signaling in regulating many aspects of normal or pathologic immune responses started about thirty years ago (1). Indeed, the discoveries of the JAK tyrosine kinases and STAT transcription factors (1) quickly unraveled that their function is required to transmit signal through type 1 and type 2 cytokine receptors (2). Since then, targeting intracellular signaling with Janus kinase (JAK) inhibitors has become a major and successful approach to treat many autoimmune and inflammatory diseases (3) with multiple JAK inhibitors approved or in late-stage clinical development for a wide array of such diseases. This short collection of articles further exemplifies the breadth and depth of the science being pursued to further advance the understanding of JAK/STAT signaling and the effects JAK inhibition in diseases as diverse as rheumatoid arthritis, inflammatory bowel disease, alopecia areata, multiple sclerosis and Sjögren’s syndrome, Behçet’s syndrome and vasculitis.

In this Frontiers in Immunology collection of articles, we have collected original works and reviews that provide new insights into the role of JAK/STAT signaling in various systems by using disruption of the signaling pathways with various pharmacological agents.

The work of Gopalakrishnan et al. explores the role of JAK/STAT signaling in intestinal epithelial cells (IECs) from colonoids, from UC patients or non-IBD controls, and how it regulates TNF+Poly(I:C)-dependent upregulation of MHC-II expression. This is a good example illustrating how JAK/STAT signaling in non-immune cells could impact the adaptive immune response *via* antigen presentation.

The review by Watanabe and Hashimoto covers the pathophysiology of large vessel vasculitis, a severe inflammatory condition of the blood vessels. Starting from the studies done in patients with rheumatoid arthritis, and the fact that two diseases are driven by similar cytokines such as IL-6, IL-12 and type I and type II IFNs, the authors report about a preclinical model of vascular inflammation as well as few case reports in which JAK inhibitors have been demonstrated to decrease vascular inflammation. In particular, the data from the human studies, albeit with the caveat that have been collected in a limited number of patients, seems to suggest that JAK inhibitors could be employed to induce remission and to reduce the use of steroids.

Zhao et al reported a case series of 4 patient with Behcet’s disease involving the gastrointestinal tract, successfully treated with tofacitinib. The intestinal involvement in Behcet’s disease may be challenging and the treatment is not yet ideal, with some patients failing to achieve endoscopic remission with immunosuppressants and biological drugs (TNF inhibitors). All four patients had failed previous treatment with different immunosuppressive drugs and, after starting tofacitinib, showed clinical and histologic improvement that allowed them to reduce the dose of glucocorticoids.

The study reported by Hong et al. compared data collected from genome-wide association studies (GWAS) and from the Gene Expression Omnibus (GEO) database to assess susceptibility genes and differentially expressed genes (DEGs) in multiple sclerosis and Sjögren’s syndrome. Interestingly, the authors found that among common susceptibility genes, several were associated with cytokines and the JAK/STAT signaling pathway. With this information in hand, the authors took advantage of the Comparative Toxicogenomics Database (CTD), DrugBank database, and Drug–Gene Interaction (DGI) Database to search for drugs that could be of potential use in these pathologies. Notably, over 130 drugs, including JAK inhibitors were found to be potentially useful in multiple sclerosis and Sjögren’s syndrome.

Given their pleiotropic effects on immune and non-immune cells involved in the pathogenesis of rheumatoid synovitis and bone erosion, JAK inhibitors have been approved for Rheumatoid Arthritis for 10 years (4). Despite the remarkable effect of this class of drugs in many patients, still not all of them respond to the treatment. Looking for biomarkers of treatment response, Tucci et al. evaluated by flow cytometry the monocyte phosphorylation of STAT1, in basal conditions and after stimulation with IL-2, IFN-α, and IL-6, in a cohort of rheumatoid arthritis patients treated with baricitinib. The authors observed a significant reduction in monocyte number and cytokine induced STAT1 phosphorylation only in responder patients, suggesting that these changes could be an early predictor of treatment response.

Palmroth et al. studied the signaling effects of the JAK1/3 inhibitor tofacitinib in a cohort of patients with rheumatoid arthritis. They focused on the levels of constitutive and cytokine-induced phosphorylated STATs in monocytes, T cells and B cells isolated from peripheral blood. They found that tofacitinib improves rheumatoid arthritis and suppresses JAK-STAT signaling. The degree of the inhibition of STAT phosphorylation by the JAK inhibitor was dependent on the cytokine used for STAT activation and differences were also found in the indicated cell types studied. Based on their findings in a small sample size the authors speculate that the assessment of the baseline JAK-STAT signaling profile may have a prognostic value for the treatment responses to certain JAK inhibitors.

The review of Lensing and Jabbari tackles the pathogenesis of alopecia areata discussing the involvement of cytokine whose signaling relies on JAK/STAT and the rationale for targeting this pathway with first- and second-generation JAK inhibitors. In particular, the authors discussed the benefits and potential limits of JAK inhibitors in patients with alopecia areata.

Yu et al. report a case on the successful treatment of alopecia universalis in a patient who received the JAK1/3 inhibitor tofacitinib. The authors documented the clinical response and the levels of some cytokines in the patient’s peripheral blood. Interestingly, they observed a strong increase in serum cytokine levels of most factors studied. So far, little data is available in the literature on serum cytokine levels during the treatment with JAK inhibitors like tofacitinib. In case there is accumulating data with similar observations in future, we need to understand why increasing cytokine levels in the blood are associated with a clinical improvement of an autoimmune disease like alopecia areata and if increased cytokine release is harmful for patients.

The basic research study by Dai et al. focused on the immunological effects of the JAK1/3 inhibitor ifidancitinib in a model of alopecia areata using C3H/HeJ mice. The authors studied the effects of ifidancitinib on cytokine-induced STAT activation in T cells and on T cell proliferation *in vitro* and *in vivo*. Ifidancitinib treatment reversed alopecia areata in C3H/HeJ mice. By elegant immunological analysis they describe that JAK1/3 inhibition regulates T cell exhaustion and suggest that this JAK/STAT-depending mechanism is responsible for the beneficial effects observed in mice with alopecia areata treated with ifidancitinib.

Finally, Iglesias et al. review recent developments on T cell costimulation blockade in treatment of transplant rejection. The focus is on mechanisms that limit CTLA-4-Ig efficacy, and it gives a thorough insight on potential complementary therapies, including JAK inhibitors to overcome the inflammation induced resistance.

## Author contributions

All authors listed have made a substantial, direct and intellectual contribution to the work, and approved it for publication.
